# Editorial: Mycoviruses of pathogenic fungi: the current research landscape

**DOI:** 10.3389/ffunb.2024.1404961

**Published:** 2024-04-12

**Authors:** Muhammad Faraz Bhatti, Ioly Kotta-Loizou, Robert H. A. Coutts

**Affiliations:** ^1^ Atta-ur-Rahman School of Applied Biosciences, National University of Sciences & Technology, Islamabad, Pakistan; ^2^ Department of Clinical, Pharmaceutical and Biological Science, School of Life and Medical Sciences, University of Hertfordshire, Hatfield, United Kingdom; ^3^ Department of Life Sciences, Faculty of Natural Sciences, Imperial College London, London, United Kingdom

**Keywords:** mycovirus, plant pathogenic fungi, virome, virus-host interaction, RNA silencing

This Frontiers Research Topic on “*Mycoviruses of pathogenic fungi: the current research landscape*” has been initiated in the context of *Frontiers in Cellular and Infection Microbiology* and *Frontiers in Fungal Biology*, covering the section *Fungal Pathogenesis*. Our Research Topic includes two high-quality Original Research manuscripts and one comprehensive Review manuscript that provide insight into the diversity and biology of mycoviruses infecting plant pathogenic ascomycetes, including the discovery and characterization of numerous mycoviruses with double-stranded (ds) and single-stranded (ss) RNA genomes belonging to established and proposed virus families, the effects of mycovirus co-infection on the morphology, growth, sporulation and virulence of the host fungus, and the potential role of mycovirus infection and host RNA silencing in biological control applications against fungal diseases in plants.

In the Original Research article “*Mycovirus hunting revealed the presence of diverse viruses in a single isolate of the phytopathogenic fungus Diplodia seriata from Pakistan*”, Khan et al. reported the diverse virome present in *D. seriata* strain L3 that was isolated from diseased leaves of the Lokath plant. *Diplodia* spp. are phytopathogenic ascomycetes known to cause fruit rot, leaf spot, canker, leaf chlorosis, and trunk dieback in many woody and herbaceous plants; however, the virome of this genus is less explored. The genomic characterization of these mycoviruses was carried out by next-generation sequencing (NGS) of viral RNA. The presence of mycoviruses corresponding to virus-like contigs was confirmed *via* reverse transcription followed by polymerase chain reaction (RT-PCR) amplification of total RNA with virus-specific primers, and terminal sequences were confirmed by RNA ligase-mediated rapid amplification of complementary (c)DNA ends (RLM-RACE). This study revealed that *D. seriata* strain L3 is co-infected with eight mycoviruses belonging to seven different viral families: the established families *Chrysoviridae*, *Partitiviridae*, *Polymycoviridae* and *Totiviridae* of dsRNA viruses, and *Botourmiaviridae* of ssRNA viruses, and the proposed families “Ambiguiviridae” and “Splipalmiviridae”. Based on their high degree of sequence diversity and according to the criteria of the International Committee on Taxonomy of Viruses (ICTV), five of the reported mycoviruses represented new species. To assess the biological impact of these viruses, curing attempts through hyphal tipping resulted in the elimination of three viruses from the original strain: Diplodia seriata polymycovirus 1, Diplodia seriata ambiguivirus 1 and Diplodia seriata splipalmivirus 1. The morphological comparison of the virus-infected and partially cured strains showed restoration of colony growth; however, it remains unknown how the eliminated mycoviruses contribute individually to host phenotypic alterations. Considering that some of these mycoviruses belong to newly established or proposed virus families for which biological information is lacking, studies focusing on mycovirus co-infections may provide a platform for further investigation of possible antagonistic or synergistic virus-host and virus-virus interactions.

The Original Research article “*Novel and diverse mycoviruses co-infecting a single strain of the phytopathogenic fungus Alternaria dianthicola*” by Zhong et al., is an excellent virome analysis of *A. dianthicola* strain HNSZ-1, which belongs to a fungal genus known to have worldwide distribution and to cause devastating disease to plants, humans and animals. Using a combination of RT-PCR, cDNA cloning and NGS, the sequences of nine co-infecting mycoviruses were determined. Five of these mycoviruses were novel, belonging to the established families *Totiviridae* of dsRNA viruses, *Hypoviridae* of positive-sense (+) ssRNA viruses, *Mymonaviridae* of negative-sense (–) ssRNA viruses and a proposed family “Ambiguiviridae”. This report complements several other studies on the viromes of plant pathogenic fungi and illustrates how multiple mycovirus infections can be tolerated by the host fungus with moderate effects on growth, sporulation and virulence. This was further confirmed by eliminating mycoviruses and investigating horizontal transmission, followed by biological comparisons that revealed that Alternaria dianthicola negative-stranded RNA virus 1 may be associated with phenotypic alterations to the host fungus.

In their Review “*Mycovirus-encoded suppressors of RNA silencing: Possible allies or enemies in the use of RNAi to control fungal disease in crops*”, Rodriguez Coy et al. argue that the arms race between mycoviruses and their host fungi during infection may have consequences for the biological control of fungal diseases in plants beyond any direct effect of mycovirus infection on fungal pathogenicity leading to, for instance, hypovirulence. Cross-kingdom RNA interference (RNAi) occurs naturally when a pathogenic fungus generates small interfering RNAs (siRNAs) that are transferred into plants to suppress their immunity and aid fungal infection. New approaches to crop protection against fungi include host- and spray-induced gene silencing (HIGS and SIGS), both of which rely on the fungal RNA silencing machinery to target key fungal pathogenicity genes as guided by RNA either produced by transgenic host plants or delivered externally by spraying. RNA silencing is also an antiviral mechanism that recognizes and acts against mycovirus infection in fungi. Consequently, mycoviruses often encode proteins with the ability to suppress RNA silencing, weakening the antiviral response of the fungus but also influencing the efficiency of cross-kingdom RNAi, HIGS and SIGS. In light of the above, the infection status of pathogenic fungi should be considered when designing biological control strategies based on fungal RNA silencing.

As Associate Editors, we would like to take this opportunity to acknowledge all the contributing authors who have chosen our Research Topic on “*Mycoviruses of pathogenic fungi: The current research landscape*” as a vehicle to share their stimulating work.

## Author contributions

MB: Writing – original draft, Writing – review & editing. IK: Writing – original draft, Writing – review & editing. RC: Writing – original draft, Writing – review & editing.

